# Perioperative Administration of an Intravenous Beta-Blocker Landiolol Hydrochloride in Patients with Lung Cancer: A Japanese Retrospective Exploratory Clinical Study

**DOI:** 10.1038/s41598-019-41520-7

**Published:** 2019-03-26

**Authors:** Atsuhiro Sakamoto, Kaori Yagi, Tatsuaki Okamura, Tomohiro Harada, Jitsuo Usuda

**Affiliations:** 1 Anesthesiology, Nippon Medical School Hospital, Tokyo, Japan; 2 Anesthesiology, Hospital Nakamura, Tokyo, Japan; 3 Clinical Development Planning II, Ono Pharmaceutical Co., Ltd, Osaka, Japan; 4 Thoracic Surgery, Nippon Medical School Hospital, Tokyo, Japan

## Abstract

Beta-blockers have been reported to improve prognosis for various cancers, but the usefulness of perioperative administration remains unclear. To assess the efficacy of perioperative administration of landiolol hydrochloride, an intravenous beta-blocker, for lung cancer, we conducted a single-center, retrospective study. This study included patients who participated in a research conducted by Nippon Medical School Hospital from August 2012 to November 2013. The main selection criteria were males and females younger than 85 years old who have undergone anatomic lung resection for lung malignancies. Fifty-seven patients, 28 in the landiolol group and 29 in the control group, were included. The postoperative relapse-free survival rate at 2 years was 0.89 (95% CI, 0.78–1.01) in the landiolol group and 0.76 (95% CI, 0.60–0.91) in the control group (Chi-squared test; *P* = 0.1828). The relapse-free survival rate tended to be higher in the landiolol group than in the control. Hazard ratio for relapse-free survival in the landiolol group compared to the control was 0.41 (95% CI, 0.13–1.34), demonstrating that relapse free survival was prolonged in the landiolol group (log-rank test; *P* = 0.1294). It was suggested that relapse-free survival was prolonged when landiolol hydrochloride was administered from the induction to completion of anesthesia. Further studies are needed to confirm our findings.

## Introduction

Throughout the world, including the United States and Japan, there are many patients with lung cancer, which has a high mortality rate. A total of 239,320 patients were suffering from lung cancer and 161,250 patients died due to the disease in the United States in 2010^1^. In Japan, 128,700 patients are predicted to be suffering from lung cancer, and the annual number of deaths is expected to be 78,000 in 2017, which is the highest among deaths due to carcinomas^2^.

Surgery is considered the main treatment for lung cancer in patients with clinical stage IIIA or less severe stages, whereas radiotherapy or chemotherapy is attempted for those with stage IIIB or more severe stages^3^. Postoperative relapse rate remains high even after undergoing radical surgery. Circulating tumor cells are reportedly dispersed during surgery^4^ and are associated with early relapse after lung cancer surgery.

Administration of human atrial natriuretic peptide (hANP), a drug for heart failure, for 3 days during the perioperative period inhibited adhesion of the dispersed circulating tumor cells to vascular endothelial cells and suppressed relapses after lung cancer surgery^5^.

Activation of the sympathetic nerves is supposed to promote tumor invasion, migration to blood vessels, and angiogenesis and thereby increase the possibility of cancer metastases and relapses^6^. Chronic administration of oral beta-blockers reportedly improved prognoses of various carcinomas^7–13^.

Landiolol hydrochloride is an intravenous, short-acting beta-blocker which has been manufactured and marketed by Ono Pharmaceutical Co., Ltd.^14–17^. According to study reports of oral beta-blockers and hANPs, we considered that administration of landiolol hydrochloride during lung cancer surgery might suppress relapses and metastases.

We previously reported that administration of landiolol hydrochloride in lung cancer patients during the perioperative period significantly suppressed atrial fibrillation postoperatively^18^. Here, we have conducted a retrospective, exploratory clinical study to evaluate effects of landiolol hydrochloride on relapses and metastases if it is administered during the perioperative period.

## Results

The analysis set consisted of 57 patients, excluding 7 patients without lung cancer or with metastatic lung tumor.

Landiolol hydrochloride was administered to 28 patients (hereinafter, the landiolol group) and placebo was administered to 29 patients (hereinafter, the control group). The dose of landiolol hydrochloride was 2.5 μg/kg/min and was administered from induction to completion of anesthesia.

Patient backgrounds of the landiolol group and control group were as follows respectively: Age (mean ± SD), 70.3 ± 7.5 years old and 69.1 ± 8.4 years old; Male, 15 patients and 17 patients; Stage IA, 16 (57.1%) and 14 (48.3%) patients; Stage IB, 3 (10.7%) and 7 (24.1%) patients; Stage IIA, 5 (17.9%) and 4 (13.8%) patients; Stage IIB, 1 (3.6%) and 3 (10.3%) patients; Stage IIIA, 2 (7.1%) and 1 (3.4%) patients; Stage IIIB, 1 (3.6%) and 0 (0.0%) patients (Table 1). Adjuvant chemotherapy was performed for 6 patients in the landiolol group and 2 patients in the control group. No differences were found in sex, age, and body mass index between both groups.

**Table 1:** Patient background.

	Landiolol group	Control group
	N = 28	N = 29
Mean age (SD)	70.3 (7.5)	69.1 (8.4)
Sex		
Male (%)	15 (53.6)	17 (58.6)
Female (%)	13 (46.4)	12 (41.4)
p-Stage		
IA (%)	16 (57.1)	14 (48.3)
IB (%)	3 (10.7)	7 (24.1)
IIA (%)	5 (17.9)	4 (13.8)
IIB (%)	1 (3.6)	3 (10.3)
IIIA (%)	2 (7.1)	1 (3.4)
IIIB (%)	1 (3.6)	0 (0.0)
Mean weight, kg (SD)	57.2 (8.4)	54.5 (10.1)
Mean BMI, kg/m^2^ (SD)	22.5 (2.6)	21.3 (3.0)
Adjuvant chemotherapy		
(+) (%)	6 (21.4)	2 (6.9)
(−) (%)	22 (78.6)	27 (93.1)

BMI, body mass index.

**Figure 1:**
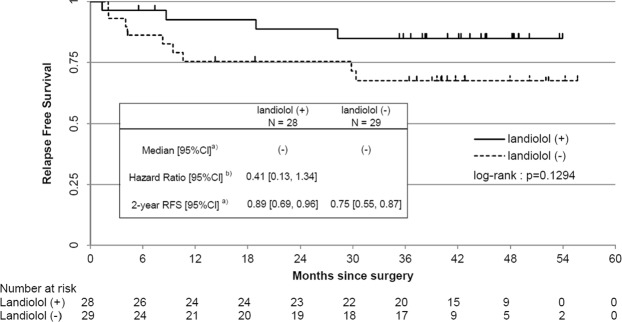
Relapse-free survival period, hazard ratio, and 2-year relapse-free survival rate in the landiolol group and control group. CI, Confidence interval; RFS, Relapse-free survival. (**a**) Estimated based on Kaplan-Meier method. (**b**) Estimated based on Cox-Proportional Hazards Model assuming treatment groups to be single factors.

The postoperative relapse-free survival rate at 2 years was 0.89 (95% CI, 0.78–1.01) in the landiolol group and 0.76 (95% CI, 0.60–0.91) in the control group (Table 2). Compared with the control group, the relapse-free survival rate tended to improve in the landiolol group (Chi-squared test; *P* = 0.1828). Hazard ratio for relapse-free survival among the landiolol group compared to the control group was 0.41 (95% CI, 0.13–1.34), which demonstrates that relapse-free survival tends to be prolonged among the landiolol group (Fig. 1 and Table 2, log-rank test; *P* = 0.1294). Hazard ratio for overall survival among the landiolol group compared to the control group was also 0.25 (95% CI, 0.03–2.26), which demonstrates similar trends (Fig. S1 and Table S1, log-rank test; *P* = 0.1836).

**Table 2:** Analysis on relapse-free survival.

	Landiolol group	Control group
	N = 28	N = 29
	n (%)	n (%)
Analysis of relapse-free survival		
Event	4 (14.3)	9 (31.0)
Censor	24 (85.7)	20 (69.0)
Median [95% CI]^a^	(−)	(−)
Log-rank test^b^	*P* = 0.1294	
Hazard ratio^c^	0.41	
[95% CI]^c^	[0.13–1.34]	
Postoperative relapse-free survival rate at 2 years^a^	0.89	0.75
[95% CI]^a^	[0.69–0.96]	[0.55–0.87]
Analysis of postoperative relapse-free survival at 2 years^d^		
Event	3 (10.7)	7 (24.1)
Censor	25 (89.3)	22 (75.9)
Postoperative relapse-free survival rate at 2 years	0.89	0.76
[95% CI]	[0.78–1.01]	[0.60–0.91]
Chi-squared test^e^	*P* = 0.1828	

Units for duration are months, and 1 month is defined as 30.4375 days.

^a^Estimated based on Kaplan-Meier method.

^b^*P < 0.05, N.S.: *P* ≥ 0.05.

^c^Estimated based on Cox-Proportional Hazards Model assuming treatment groups to be single factors.

^d^Events occurred at least 2 years after surgery in 1 patient in the landiolol group and 2 patients in the control group, and these subjects were handled as not having experienced events.

^e^*P < 0.05, N.S.: *P* ≥ 0.05.

As adjuvant chemotherapy was administered in 6 patients (21.4%) in the landiolol group and 2 patients (6.9%) in the control group, this difference might have affected recurrence. Therefore, we explored the relapse-free survival of those subjects without adjuvant chemotherapy (Table 3). The recurrence rate was 18.2% (4/22) in the landiolol group and 33.3% (9/27) in the control group [log-rank test; *P* = 0.2363, HR = 0.50 (95% CI, 0.15–1.62)].

**Table 3:** Analysis on relapse-free survival in subjects without adjuvant chemotherapy.

	Landiolol group	Control group
	N = 22	N = 27
	n (%)	n (%)
Event	4 (18.2)	9 (33.3)
Censor	18 (81.8)	18 (66.7)
Log-rank test^a^	*P* = 0.2363	
Hazard ratio^b^	0.50	
[95% CI]^b^	[0.15–1.62]	

^a^*P < 0.05, N.S.: *P* ≥ 0.05.

^b^Estimated ^b^ased on Cox-Proportional Hazards Model assuming treatment group to be single factor.

## Discussion

Previous studies state that oral beta-blockers improved prognosis for various cancers^7–13^. The anti-tumor effect of beta-blockers may be mediated by suppression of the sympathetic nervous system via blocking of catecholamines^6^. During surgery, a great amount of catecholamine is secreted, leading to an activated state of the sympathetic nerves, so it may be beneficial for the patient undergoing cancer surgery if activation of the sympathetic nervous system is suppressed by administration of beta blockers. Beta-blockers were reported to show a more positive effect on OS in patients who underwent surgery (HR = 0.60, 95% CI: 0.42–0.86) than in those who did not undergo surgical treatment (HR = 0.80, 95% CI: 0.69–0.93), based on meta-analysis of 12 studies^19^. While in the previous studies beta blockers were administered long-term, in our study landiolol was administered only during the perioperative period. As in our study, a phase II, placebo-controlled clinical trial suggested that short-term administration of propranolol and etodolac (a COX-2 inhibitor) from 5 days before surgery to 5 days after surgery appeared to improve the three-year recurrence rate in patients with colorectal cancer^20^. In that study, recurrence rates were 26.3% (5/19) in the placebo group and 6.7% (1/15) in the treatment group (*P* = 0.35). They explained that neuroendocrine and paracrine stress-inflammatory responses due to surgery may promote progression of micro-metastases. In addition, administration of human atrial natriuretic peptide (hANP), a drug used for heart failure, for 3 days during the perioperative period improved RFS in patients with lung cancer^5^. In this paper, hANP suppressed inflammation due to surgery, preventing adhesion of the dispersed circulating tumor cells to vascular endothelial cells. Although we have not examined the mechanism of the putative anti-relapse effect of landiolol, landiolol may also inhibit attachment of circulating tumor cells to vascular endothelial cells for the following reasons: activation of the sympathetic nervous system is suppressed, tumor cells are dispersed during lung cancer resection^4^, and adhesion of cancer cells to vascular endothelial cells is promoted by norepinephrine and suppressed by propranolol^21^.

Generally, most cardiovascular drugs taken before surgery are discontinued to ensure the patient’s safety during surgery. Lung cancer surgery in particular is performed using one-lung ventilation, so patients have added stress to their left heart during surgery. Preoperative treatment using beta-blockers thus carries the risk of causing adverse effects on circulatory dynamics such as hypotension and bradycardia, influencing the safety of lung surgery. Landiolol has an unusually short half-life (about 4 minutes) and dosage is very easily adjusted. Furthermore, landiolol has extremely high selectivity for the beta-1 receptor, with a Ki for beta receptors (nmol/L) of 62.1 ± 10.0 (mean ± SD) for the beta-1 receptor and 1890 ± 80 (mean ± SD) for the beta-2 receptor^22^. Because effects on beta-2 receptors may cause bronchoconstriction, high selectivity for beta-1 receptors may contribute to its safety. Landiolol is already used widely and safely in patients with tachyarrhythmias during surgery where cardiac dysfunction is present^14–17^. Hence, landiolol is thought to be safer than other, orally administered beta-blockers in this clinical setting.

In addition, this mechanism is unlikely to depend on the type of carcinoma because it was found to suppress activation of sympathetic nerves in a host when an intravenous beta-blocker was used for a very short time during cancer surgery. Therefore, this therapy may be applicable to any radically resectable cancer.

There was however a difference in the usage of adjuvant chemotherapy in our study, so we evaluated relapse-free survival in subjects without adjuvant chemotherapy (Table 3). Although there was no significant difference, the hazard ratio was 0.50 (95% CI, 0.15–1.62), we therefore think that further studies are needed to evaluate the efficacy of landiolol. It should be noted that an investigator-initiated trial is being conducted (Premium Land study; clinical trial registry No. jRCT2011180004).

In addition, we conducted some ad-hoc analysis and found that the hazard ratio of relapse-free survival adjusted by sex, age, BMI and cancer stage was 0.15 (95% CI: 0.03–0.83, *P* = 0.030) (Table S2). These results could be due to the deviation of cancer stage in patients with events, or 4 patients with events in landiolol group including 2 patients with stage 3 lung cancer, and 9 patients with events in control group including 1 patients with stage 3 lung cancer. Therefore, we conducted sub-analysis in only stage 1 and 2 patients, and found that the relapse-free survival more tended to be prolonged among the landiolol group by Kaplan-meier method (log-rank test; *P* = 0.0503). (Fig. S2 and Table S3).

There are several limitations to this study. Firstly, this is a retrospective study focused on a single center and a small sample size. On the other hand, given that the previous study was a prospective randomized controlled trial, there are no variations in patient background and no selection bias for patients. Secondly, while the previous study had no rules in terms of adjuvant chemotherapy, treatment was administered according to the Lung Cancer Treatment Guidelines. Thirdly, the follow-up time was not long enough to allow us to fully address long-term survival outcomes.

## Conclusions

Administering landiolol hydrochloride at low doses during cancer surgery for a short period of time tends to improve relapse-free survival rate as well as prolong relapse-free survival and overall survival. This study was a retrospective, exploratory clinical study in a small sample size; it would be beneficial if large-scale, prospective clinical studies are conducted in the future.

## Methods

### Data collection

After obtaining approval for the study from the ethics committee of Nippon Medical School, a retrospective study based on the medical records of 64 patients who participated in the previous clinical trial to prevent atrial fibrillation after lung cancer surgery^13^ (UMIN000007561) was performed. These subjects included 54 patients who were considered the analysis set of the previous study and underwent lung cancer surgery between August 2012 to September 2013, as well as 10 additional patients who participated in the same study by November 2013. Informed consent from patients was obtained through an opt-out method. Patients did not receive a stipend.

This clinical study targeted males and females younger than 85 years old who have undergone anatomic lung resection for lung malignancies and had a physical status classification of 1–2 based on the American Society of Anesthesiologists. Those participating in the study were randomly assigned to either landiolol hydrochloride (landiolol group) or placebo (control group), and 2.5 μg/kg/min was administered for the landiolol group from induction to completion of anesthesia. Staging of lung cancer were determined by the classification at that time.

### Statistical analysis

Postoperative relapse-free survival rate at 2 years was calculated for each group using the Kaplan-Meier method, and two-sided 95% confidence interval (CI) was also calculated. Chi-square tests were performed to compare the treatment groups.

The Kaplan-Meier curve for relapse-free survival and overall survival was displayed for each treatment group. In addition, the median and two-sided 95% CI were calculated using the Kaplan-Meier method. Log-rank tests were used to compare the treatment groups. The hazard ratio and two-sided 95% CI for the landiolol group compared to the control group was calculated using the Cox-Proportional Hazards Model with treatment groups being the single factor. Significance was defined at the level of P < 0.05. All data were statistically analyzed using Stata version14 (Stata Corp, College Station, TX).

Relapse was defined as follows. Relapse included those diagnosed based on imaging diagnostics as well as those diagnosed based on exacerbation of symptoms not dependent on imaging diagnostics (i.e. clinical relapse). If relapse was diagnosed based on imaging diagnostics, the day the test was performed was considered the date of relapse, whereas date of relapse for clinical relapse was defined as the day relapse was diagnosed. Periods where tumor markers alone increased were not considered relapse, and relapse was only defined when imaging diagnostic tests confirmed relapse or when clinical relapse was determined based on exacerbation of symptoms. Survival cases where relapse was not confirmed were censored on the day of final confirmation of relapse-free survival. If relapse was diagnosed based on imaging diagnostics, relapse events were only considered on the “day of imaging diagnostic tests” that provided a “confirmed diagnosis” and not the day of tests resulting in “suspected relapse due to imaging”. If relapse was determined clinically and not based on imaging diagnostics, relapse events were considered on the day relapse was determined. If confirmed diagnosis was based on pathology diagnosis using biopsies, relapse events were defined as the day of clinical diagnosis if clinical relapse was diagnosed prior to biopsy and or as the day the biopsy was conducted if clinical relapse was diagnosed based on pathology diagnosis of biopsies without a prior clinical diagnosis. Occurrence of secondary cancer, multiple metachronous primary cancers, and multiple metachronous cancers were considered to be neither events nor censors, and patients were considered to be relapse-free until other events were observed.

## Supplementary information

Supplementary information
