# Supplementary material for: Perioperative Administration of an Intravenous Beta-Blocker Landiolol Hydrochloride in Patients with Lung Cancer: A Japanese Retrospective Exploratory Clinical Study
Source: Sci Rep. 2019 Mar 26;9:5217. doi: 10.1038/s41598-019-41520-7 (PMC6435747; doi:10.1038/s41598-019-41520-7)
Supplement: Supplementary file 1 — Supplementary information [file 41598_2019_41520_MOESM1_ESM.docx]

**Perioperative Administration of an Intravenous Beta-Blocker Landiolol Hydrochloride in Patients with Lung Cancer**: **A Japanese Retrospective Exploratory Clinical Study**

Atsuhiro Sakamoto^1,*^, Kaori Yagi^2^, Tatsuaki Okamura^3^, Tomohiro Harada^3^, and Jitsuo Usuda^4^

^1^Anesthesiology, Nippon Medical School Hospital, Tokyo, ^2^Anesthesiology, Hospital Nakamura, Tokyo, ^3^Clinical Development Planning II, Ono Pharmaceutical Co., Ltd., Osaka, and ^4^Thoracic Surgery, Nippon Medical School Hospital, Tokyo, Japan

**Supplementary information**

**Table S1.** Analysis on overall survival

|  | Landiolol group | Control group |
| --- | --- | --- |
|  | N = 28 | N = 29 |
|  | n (%) | n (%) |
| Event | 1 (3.6) | 4 (13.8) |
| Censor | 27 (96.4) | 25 (86.2) |
| Median [95% CI]^a)^ | (–) | (–) |
| Log-rank test^b)^ | *P* = 0.1836 |  |
| Hazard ratio^c)^ | 0.25 |  |
| [95% CI]^c)^ | [0.03–2.26] |  |

Units for duration are months, and 1 month is defined as 30.4375 days.

a) Estimated based on Kaplan-Meier method.

b) *: *P* < 0.05, N.S.: *P* ≥ 0.05

c) Estimated based on Cox-Proportional Hazards Model assuming treatment groups to be single factors.

**Table S2.** Multivariable Cox proportional hazards model for relapse-free survival.

| Variable | Hazard Ratio^a)^ | 95%CI^a)^ | *P*^b)^ |
| --- | --- | --- | --- |
| Use of landiolol |  |  |  |
| No | Ref. |  |  |
| Yes | 0.15 | 0.03-0.83 | 0.030* |
|  |  |  |  |
| Age | 1.07 | 0.97-1.18 | 0.159 |
|  |  |  |  |
| Sex |  |  |  |
| Female | Ref. |  |  |
| Male | 1.26 | 0.26-6.20 | 0.776 |
|  |  |  |  |
| BMI | 1.06 | 0.83-1.37 | 0.629 |
|  |  |  |  |
| p-Stage |  |  |  |
| IA | Ref. |  |  |
| IB | 2.96 | 0.40-21.83 | 0.287 |
| IIA | 3.99 | 0.49-32.32 | 0.195 |
| IIB | 17.07 | 2.54-114.89 | 0.004* |
| IIIA | 48.37 | 4.58-510.51 | 0.0012* |
| IIIB | 212.76 | 8.09-5596.22 | 0.0014* |

BMI, Body Mass Index.

a) Estimated based on Cox-Proportional Hazards Model assuming treatment groups, age, sex, BMI and p-stage to be factors.

b) *: *P* < 0.05, N.S.: *P* ≥ 0.05

**Table S3.** Analysis on relapse-free survival for stage 1 and 2 patients.

|  | Landiolol group | Control group |
| --- | --- | --- |
|  | N = 25 | N = 28 |
|  | n (%) | n (%) |
| Event | 2 (8.0) | 8 (28.6) |
| Censor | 24 (85.7) | 20 (69.0) |
| Median [95% CI]^a)^ | (–) | (–) |
| Log-rank test^b)^ | *P* = 0.0503 |  |
| Hazard ratio^c)^ | 0.24 |  |
| [95% CI]^c)^ | [0.05–1.13] |  |

Units for duration are months, and 1 month is defined as 30.4375 days.

a) Estimated based on Kaplan-Meier method.

b) *: *P* < 0.05, N.S.: *P* ≥ 0.05

c) Estimated based on Cox-Proportional Hazards Model assuming treatment groups to be single factors.

**Figure S1.** Kaplan Meier curve of overall survival in the landiolol group and control group


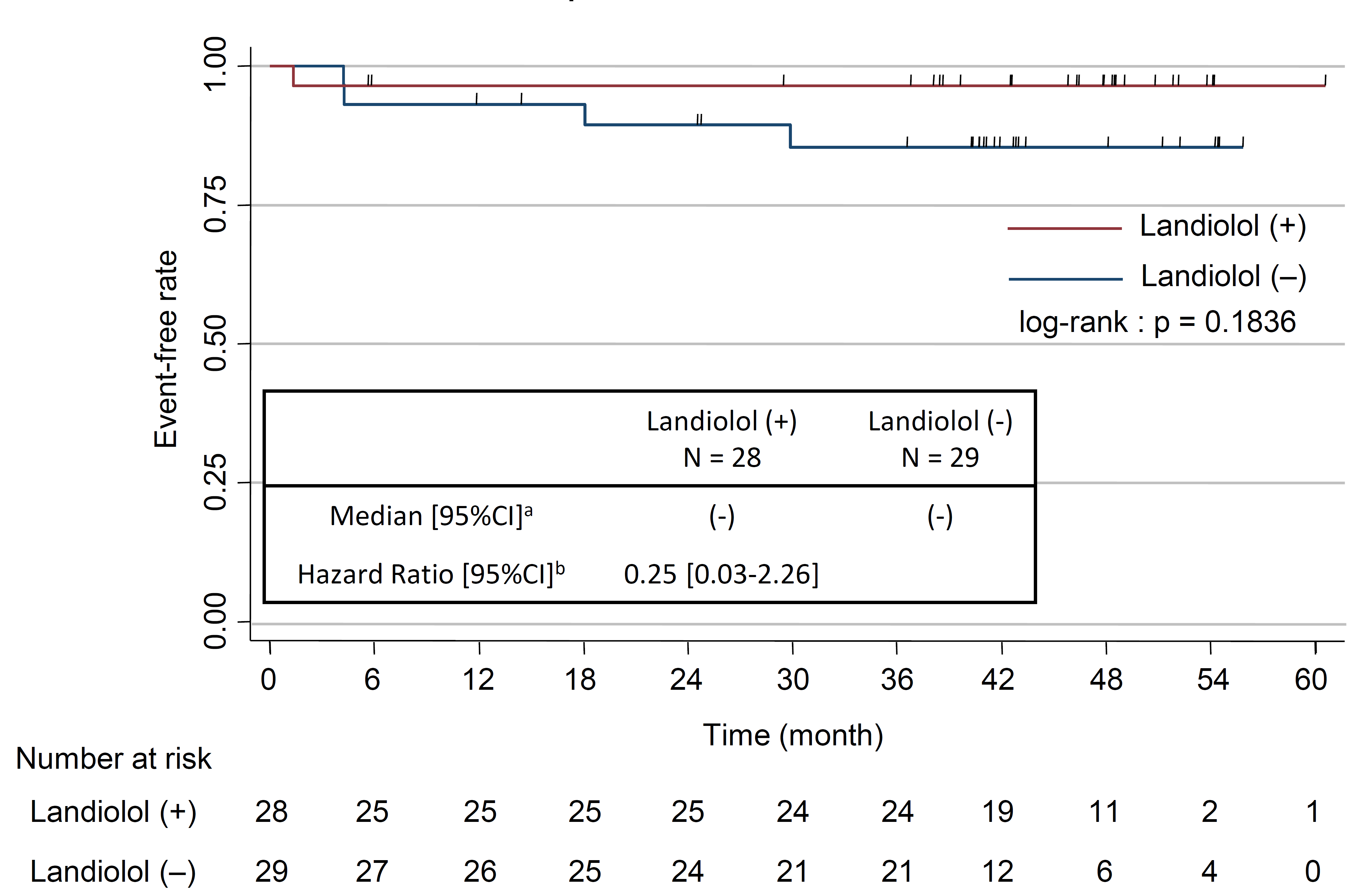


CI, Confidence interval.

a) Estimated based on Kaplan-Meier method.

b) Estimated based on Cox-Proportional Hazards Model assuming treatment groups to be single factors.


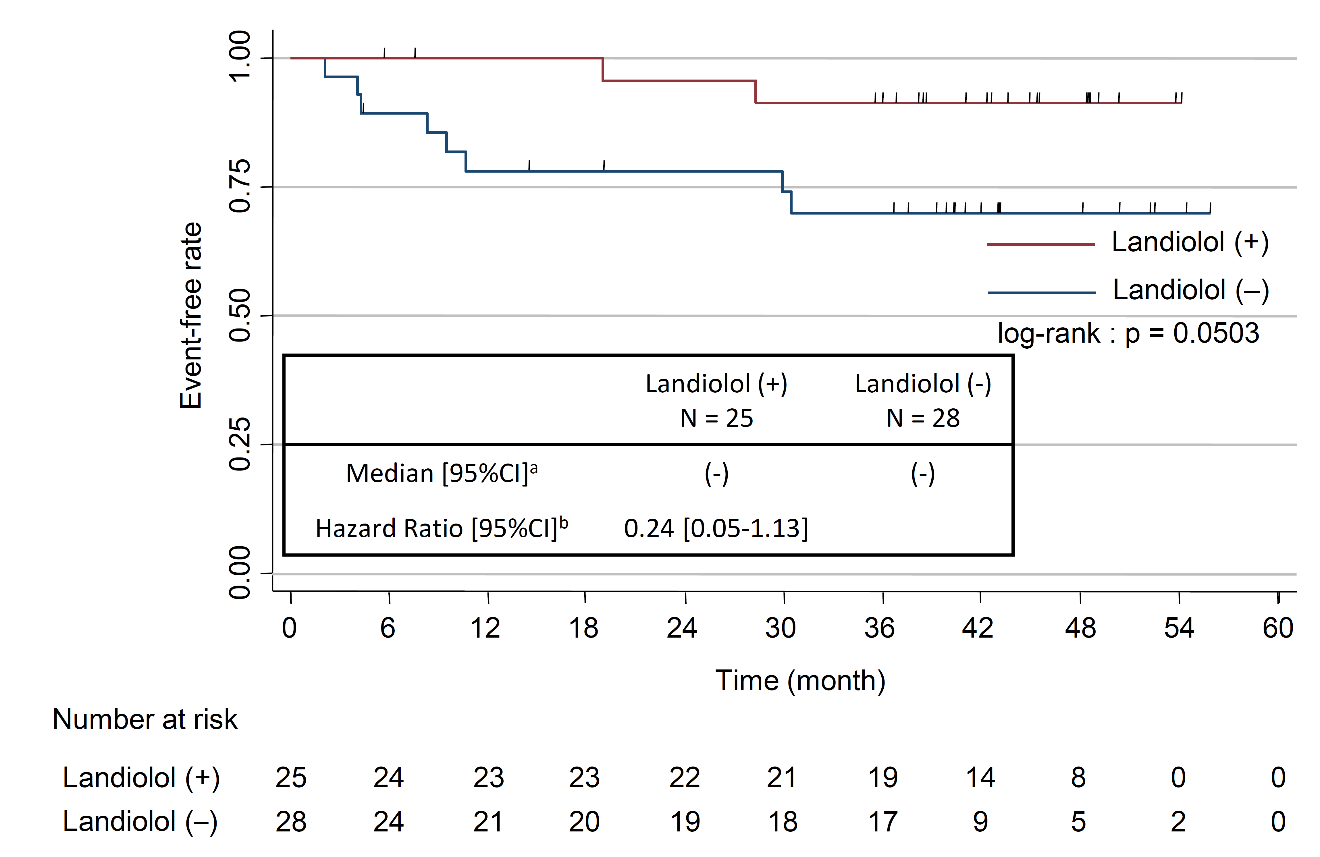
**Figure S2.** Kaplan Meier curve of relapse-free survival in the landiolol group and control group for stage 1 and 2 patients.

CI, Confidence interval.

a) Estimated based on Kaplan-Meier method.

b) Estimated based on Cox-Proportional Hazards Model assuming treatment groups to be single factors.
